# Neurosteroids and Focal Epileptic Disorders

**DOI:** 10.3390/ijms21249391

**Published:** 2020-12-10

**Authors:** Maxime Lévesque, Giuseppe Biagini, Massimo Avoli

**Affiliations:** 1 Montreal Neurological Institute-Hospital & Department of Neurology and Neurosurgery, 3801 University Street, Montreal, QC H3A 2B4, Canada; massimo.avoli@mcgill.ca; 2 Department of Biomedical, Metabolic and Neural Sciences, University of Modena and Reggio Emilia, Via Università 4, 41121 Modena, Italy; giuseppe.biagini@unimore.it; 3 Department of Physiology, McGill University, Montreal, QC H3A 2B4, Canada

**Keywords:** neurosteroids, epilepsy, allopregnanolone, allotetrahydrodeoxycorticosterone, GABA, epileptogenesis, ictogenesis

## Abstract

Neurosteroids are a family of compounds that are synthesized in principal excitatory neurons and glial cells, and derive from the transformation of cholesterol into pregnenolone. The most studied neurosteroids—allopregnanolone and allotetrahydrodeoxycorticosterone (THDOC)—are known to modulate GABA_A_ receptor-mediated transmission, thus playing a role in controlling neuronal network excitability. Given the role of GABA_A_ signaling in epileptic disorders, neurosteroids have profound effects on seizure generation and play a role in the development of chronic epileptic conditions (i.e., epileptogenesis). We review here studies showing the effects induced by neurosteroids on epileptiform synchronization in in vitro brain slices, on epileptic activity in in vivo models, i.e., in animals that were made epileptic with chemoconvulsant treatment, and in epileptic patients. These studies reveal that neurosteroids can modulate ictogenesis and the occurrence of pathological network activity such as interictal spikes and high-frequency oscillations (80–500 Hz). Moreover, they can delay the onset of spontaneous seizures in animal models of mesial temporal lobe epilepsy. Overall, this evidence suggests that neurosteroids represent a new target for the treatment of focal epileptic disorders.

## 1. Introduction

Neurosteroids are a class of compounds that modulate neuronal excitability at the level of ion channels and membrane receptors [1,2,3]. They fall into three main classes, i.e., sulfated, pregnane, and androstane neurosteroids [4]. The first class exerts excitatory effects, while the latter two play inhibitory roles [4,5]. For instance, sulfated neurosteroids can increase neuronal excitability by interacting with the picrotoxin (PTX) site of the GABA_A_ receptor [6,7] or by acting as a positive allosteric modulator of the NMDA receptor [8]. In contrast, pregnane neurosteroids, such as allopregnanolone, pregnanolone and allotetrahydrodeoxycorticosterone (THDOC), positively modulate GABA_A_ signaling [9]. Given the role of GABA-mediated activity in epileptic disorders, pregnane neurosteroids can act as broad spectrum anti-convulsants [10,11,12,13,14,15,16,17] that modulate the efficacy of GABA_A_ receptor function to enhance inhibition in the brain [18,19,20,21,22]. Allopregnanolone and THDOC are synthesized in the brain from the transformation of cholesterol into pregnenolone by the cytochrome P450 cholesterol side chain cleavage (P450scc), independently of peripheral organs such as the ovaries and adrenal glands [2]. Accordingly, P450scc is expressed in various brain regions, such as the amygdala, hippocampus, thalamus, hypothalamus, cortex, cerebellum and olfactory bulb [2,3,23,24]. P450scc is mostly located in glial cells and principal neurons, whereas its expression is limited in interneurons [2,4,11,23].

In 1986, Majewska et al. discovered that allopregnanolone is a potent allosteric modulator of GABA_A_ receptors [4,25]. In 1997, Zhu and Vicini [26] found that THDOC increases the open probability of GABA_A_ receptor channels by facilitating late channel openings, while, a few years later, it was shown that it can potentiate both GABA_A_ receptor phasic and tonic currents [10,27]. It is worth noting that the presence of the δ subunit in the GABA_A_ receptor confers greater transduction of the neurosteroids’ action [27,28]. GABA_A_ receptors containing the δ subunit are mainly found extrasynaptically, are more resistant than synaptic receptors to desensitization and have a low affinity to GABA, even at saturating concentrations [27,29]. Allopregnanolone and THDOC can enhance the tonic current generated by activating these δ GABA_A_ receptors [30,31].

We will review here recent evidence showing that neurosteroids can modulate epileptiform synchronization in vitro, as well as ictal and interictal activities in animal models of mesial temporal lobe epilepsy (MTLE) and in epileptic patients. In addition, neurosteroids appear to be able to alter the processes leading to epileptogenesis. Overall, these studies lead us to conclude that neurosteroids may represent a new therapeutic target for the treatment of human focal epileptic disorders.

## 2. Effects on 4-Aminopyridine-Induced Epileptiform Synchronization In Vitro

An early in vitro study performed in rat hippocampal slices reported that the neurosteroid allopregnanolone (5α-pregnan-3α-ol-20-one), its 5β-epimer pregnanolone and pregnenolone sulfate can modulate the spontaneous interictal-like epileptiform discharges induced in the CA3 region by the GABA_A_ receptor antagonist picrotoxin or the voltage-gated K^+^ channel blocker 4-aminopyridine (4AP) [32]. 4AP is known to indirectly enhance Ca^2+^ entry into presynaptic nerve terminals, thus increasing neurotransmitter release at both excitatory and inhibitory synapses (see for a review Avoli et al. [33]; Avoli and de Curtis [34]). In this study, Salazar et al. [32] found a concentration-dependent ability of allopregnanolone to reduce both PTX- and 4AP-induced interictal spiking while 5β-epimer pregnanolone failed to alter PTX-induced but was partially effective on the 4AP-induced activity. In addition, pregnenolone sulfate increased the frequency of both PTX-induced bursting, while in the 4AP model it increased the frequency of a specific type of interictal event [32].

**Figure 1:**
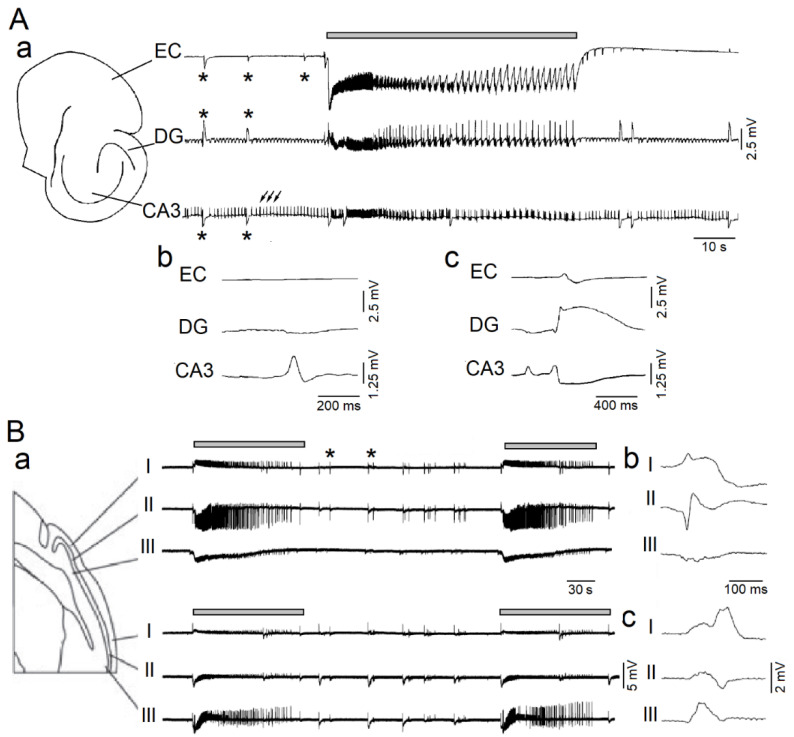
Epileptiform patterns induced by 4-aminopyridine (4AP) in rodent brain slices including the hippocampus-entorhinal cortex or the piriform cortex. (**A**): In panel (**a**), simultaneous field potential recordings obtained from the entorhinal cortex (EC), dentate gyrus (DG) and CA3 subfield of the hippocampus show the occurrence of three different types of epileptiform activity; the first (grey line)—which is recorded synchronously in all areas—consists of sustained discharge resembling a focal ictal discharge; the second type (asterisks) occurs in all areas and is characterized by slow interictal spikes; the third type (arrows) consists of continuous fast interictal-like events that are recorded in the CA3 subfield. Expanded traces of one fast and one slow interictal spike are shown in panels (**b**) and (**c**), respectively. (**B**): In panel (**a**), simultaneous field potential recordings obtained from the anterior and posterior portions of the piriform cortex in a sagittal slice; electrodes were positioned in layers I, II and III, as shown in the slice drawing. Note that both focal ictal discharges (grey lines) and interictal spikes (asterisks) occur spontaneously; two interictal spikes are expanded in panels (**b**,**c**). Panels shown in (**A**,**B**) are modified from the studies by Avoli et al. [36] and by Panuccio et al. [37], respectively.

It is well established that 4AP induces two types of spontaneous interictal discharges—which have been identified as *fast* and *slow* events—in the isolated hippocampal slice preparation [35]. The *fast* events (arrows in Figure 1A, CA3 trace) mirror the synchronous discharge of CA3 pyramidal cells that are interconnected through glutamatergic excitatory synapses, while the *slow* events (asterisks in Figure 1A, CA3 trace) mainly reflect the synchronous firing of inhibitory interneurons leading to GABA release and subsequent activation of GABA receptor subtypes. In keeping with this view, 4AP-induced *slow* interictal events disappear during GABA_A_ receptor antagonism, while they continue to occur and propagate in the absence of glutamate-mediated synaptic transmission; it has been proposed that these latter, glutamatergic-independent phenomena depend on gap-junctions and the excitatory effects caused by the concomitant occurrence of transient elevations in [K^+^] (see for review Avoli et al. [33]; Avoli and de Curtis [34]).

More complex patterns of spontaneous epileptiform discharge are, however, recorded during 4AP application in extended brain slices that include limbic or olfactory cortical structures [33,34]. As shown in Figure 1Aa (grey bar), in addition to the two types of interictal discharge (panel b and c), prolonged periods of epileptiform synchronization—which resemble ictal events—can occur in combined hippocampus-entorhinal cortex slices [36]. These ictal discharges appear to initiate in the entorhinal or perirhinal cortices as well as in the amygdala depending on the type of cut used to obtain extended brain slices that include specific areas. Moreover, both *slow* interictal and ictal discharges have been recorded from the piriform (Figure 1B, (grey bars) [37], insular [38] or cingulate cortices [39] in in vitro rodent brain slices. Finally, as reported in EEG recordings obtained in vivo from epileptic patients [40,41,42] or rodents [40,43], the epileptiform activity generated in vitro by brain slices is characterized by the occurrence of high frequency oscillations (HFOs, 80–500 Hz).

HFOs are not visible in standard field potential recordings and can only be extracted by amplifying the appropriately filtered signal. Pathological HFOs—which have been categorized according to their frequency as ripples (80–200 Hz) and fast ripples (250–500 Hz)—presumably reflect the activity of dysfunctional neural networks sustaining epileptic discharges, and they are now considered better markers than interictal spikes to identify seizure onset zones [44]. Several mechanisms may contribute to HFOs, and their exact roles remain elusive; however, it has been proposed that ripples may represent population-inhibitory postsynaptic potentials generated by principal neurons entrained by synchronously active interneuron networks [45,46]; in contrast, fast ripples should mirror in-phase or out-of-phase synchronous firing of abnormally active principal cells, and they would be independent of inhibitory neurotransmission [47,48,49,50,51].

**Figure 2:**
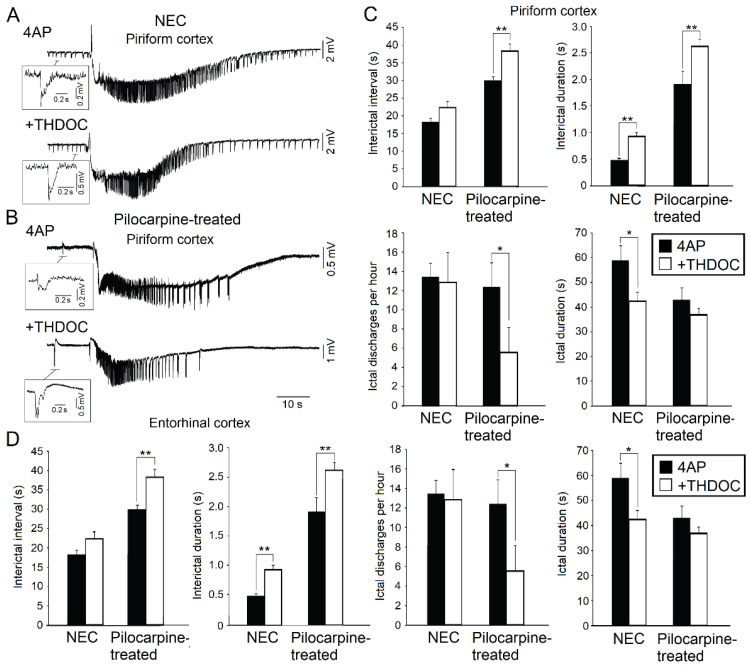
(**A**): Effects induced by allotetrahydrodeoxycorticosterone (THDOC) on the 4AP-induced epileptiform activity generated in vitro by the piriform cortex obtained from non-epileptic control (NEC) rodents. Note that both interictal (enlarged sample) and ictal discharges can be recorded during application of 4AP and following the addition of THDOC. (**B**): 4AP-induced interictal and ictal discharges recorded from the piriform cortex of brain slices obtained from epileptic (Pilocarpine-treated) rodents in 4AP and following bath application of THDOC. (**C**): Quantification of the interictal events and ictal events recorded from the piriform cortex during 4AP application and after application of THDOC in brain slices obtained from NEC and pilocarpine-treated animals. (**D**): Quantification of the interictal and ictal events recorded from the entorhinal cortex during 4AP application and after application of THDOC in brain slices obtained from NEC and pilocarpine-treated animals. * indicates *p* < 0.05; ** indicates *p* < 0.01. Panels shown in this figure are modified from the study by Shiri et al. [17].

**Figure 3:**
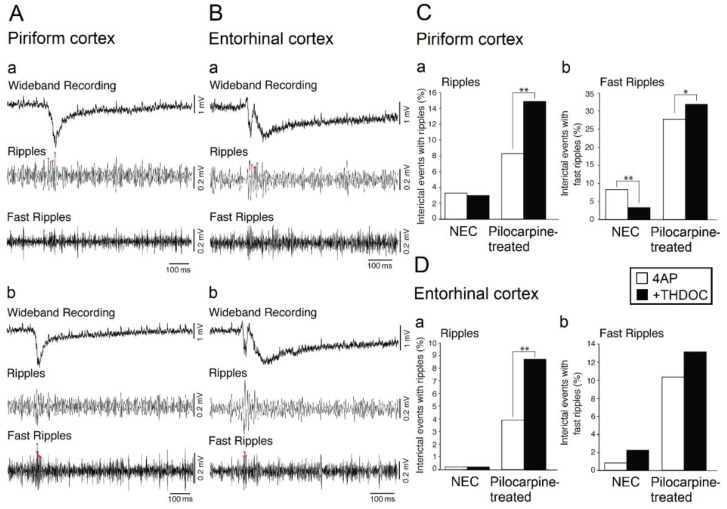
HFOs associated with 4AP-induced interictal discharges occurring in the piriform (**A**) and entorhinal cortex (**B**); field potential recordings showing interictal events co-occurring with ripples and fast ripples are shown in panels (**a**,**b**), respectively. (**C**,**D**): Quantification of the effects induced by THDOC on the ripples and fast ripples associated with 4AP-induced interictal events recorded from the piriform and entorhinal cortex of brain slices that were obtained from NEC and pilocarpine-treated rats. Note that THDOC increases the association of ripples with the interictal discharges occurring in both the piriform and entorhinal cortex of epileptic animals; THDOC also decreases and increases fast ripple occurrence in the piriform cortex of NEC and epileptic brain slices, respectively, but does not induce any significant change in fast ripples recorded from the entorhinal cortex. * indicates *p* < 0.05; ** indicates *p* < 0.01. Panels shown in this figure are modified from the study by Shiri et al. [17].

During the last few years, we have employed extended rat brain slices—which included either the piriform cortex or the entorhinal cortex—to analyze the ability of the neurosteroid THDOC to influence the epileptiform activity induced by 4AP in non-epileptic control (NEC) (Figure 2A) and pilocarpine-treated animals (Figure 2B) [15,17]. THDOC is known to enhance brain inhibition [52] and to induce anticonvulsant effects in vivo [10,19,53,54] (see also Section 3). As shown in Figure 2A,C,D, THDOC decreased the duration of the ictal discharges while prolonging the interictal spikes recorded in brain slices that had been obtained from NEC rats and included the piriform cortex or the entorhinal cortex. In addition, when analyzing ripples and fast ripples associated to 4AP-induced ictal discharges in the piriform (Figure 3A) and entorhinal cortex (Figure 3B), we found in these experiments that THDOC decreased the occurrence of fast ripples that were associated with interictal spikes (Figure 3C). THDOC, however, induced in pilocarpine-treated animals a significant increase of ripples in the piriform and entorhinal cortices and a significant increase of fast ripples in the piriform cortex (Figure 3C,D). Overall, these results demonstrate that THDOC plays a modulatory role on the epileptiform synchronization and that its effects are structure-dependent and more pronounced in epileptic than in NEC tissue.

Herrington et al. [15] also found that THDOC can induce a dose-dependent decrease in the duration of the *fast* interictal events recorded from the CA3 subfield of the hippocampus; in contrast, the duration of the *slow* interictal spikes increased. Moreover, THDOC potentiated the *slow* interictal events that were recorded during a pharmacological blockade of glutamatergic transmission but had no effect on the interictal pattern that was generated during GABA_A_ receptor antagonism. Therefore, these results suggest that potentiation of GABA_A_ receptor-mediated signaling by THDOC differentially affects *slow* and *fast* interictal discharges. It was also found in this study [15] that HFOs were modulated by THDOC. In addition, when it was applied to 4AP-treated slices, THDOC led to an increase in ripple occurrence without any concomitant change in fast ripple activity, thus indicating that it increases GABA-receptor mediated function. As already mentioned, ripples presumably reflect the synchronous activity of GABAergic interneurons [46,55,56,57]. Note that the amplitude of ripples and fast ripples associated with the interictal spikes was also increased by THDOC; such effects could reflect the increased recruitment of synchronized pyramidal cells [15], although this hypothesis needs to be confirmed.

## 3. Effects in Animal Models of Mesial Temporal Lobe Epilepsy In Vivo

In animal models of focal epilepsy, GABA_A_ receptor expression is extensively altered in a region- and neuronal-subtype-specific manner, which ultimately affects both phasic and tonic inhibitory currents [58,59]. For instance, following the induction of temporal lobe epilepsy in rats with continual hippocampal stimulation, the δ subunit-containing receptor expression is reduced in dentate gyrus granule cells, while there is an increase in the expression of α_4_γ_2_-containing subunits [60]. The increase in α_4_γ_2_-containing subunits presumably acts as a compensatory mechanism for the initial reduction in tonic inhibition. Similar findings were also observed in pilocarpine-treated mice, but these authors also found that δ subunit containing receptor expression increased in hippocampal interneurons [61]. Furthermore, dentate gyrus granule cells exhibit an increase in the expression of synaptic GABA_A_ receptors, which is coupled with a reduced sensitivity to benzodiazepines [62]. The down-regulation of the δ subunit-containing receptors suggests that temporal lobe epilepsy may be associated with reduced levels of neurosteroid sensitivity [62]. Additional mechanisms in epileptic animals that can alter neurosteroid sensitivity include a loss of GABAergic neurons in the subiculum [63,64] and a shift in inhibition such that principal cell GABAergic input is reduced, while interneuronal GABAergic input is enhanced in the piriform cortex [65].

Høgskilde et al. [66] were the first to demonstrate in vivo that a systemic pre-administration of pregnanolone could protect against acute seizures induced with several chemoconvulsants, such as pentetrazole, bicuculline, picrotoxin and strychnine. Similar results were obtained by Belelli et al. [67], who also reported that more potent anti-ictogenic effects were obtained when allopregnanolone was administered before the GABA_A_ receptor antagonist bicuculline. Allopregnanolone was, however, ineffective in the maximal electroshock model and against strychnine-induced seizures, as later confirmed by Kokate et al. [68]. Further studies also showed that allopregnanolone induces a dose-dependent protection against seizures in the 6 Hz and pentylenetetrazole model, as well as partial protection against NMDA-induced seizures [68,69,70].

The anti-ictogenic properties of neurosteroids are not only observed in acute seizure models, but also in chronic animal models of MTLE, such as in the pilocarpine and kainic acid model. Pilocarpine is a cholinergic muscarinic agonist that when systemically injected in rodents, induces a *status epilepticus* (SE) that is followed by a latent period—during which no seizures are observed—and then by recurrent, spontaneous non-convulsive and convulsive seizures [71,72]. Kainic acid—which is a cyclic analog of L-glutamate and an agonist of the ionotropic kainate receptor—when administered locally or systemically in rodents, also induces an SE that is followed by a latent period and then by the occurrence of spontaneous focal seizures that can become secondarily generalized [73,74,75,76]. Kokate et al. [70] have shown that allopregnanolone and THDOC can prevent the development of pilocarpine-induced SE in rodents. Furthermore, when administered 15 min after the onset of SE, pregnanolone completely abolished ictal activity. Saporito et al. [77] recently reported that such effects can be observed when allopregnanolone or ganaxolone (the 3*β*-methylated synthetic analog of allopregnanolone) are administered 15 min after SE onset in the lithium-pilocarpine model. Ganaxolone had similar anti-ictogenic properties when administered 60 min after SE onset, whereas only transient effects were observed with allopregnanolone [77].

Kokate et al. have also reported that pregnanolone and THDOC provide protection against the development of kainic acid-induced SE in mice [70]. However, neurosteroids had less potent anti-ictogenic effects in this model than in the pilocarpine model. According to these investigators, this difference should be due to the prolonged duration of the kainic acid-induced SE as compared to pilocarpine-induced SE; in line with their view, they found that a second administration of the investigated neurosteroid 1 h after the first dose was needed to obtain a complete anti-ictogenic effect in the kainic acid model [70].

Although neurosteroid levels can significantly modulate SE in animal models of MTLE, it was unclear until a few years ago whether they also display any relationships with the duration of the latent period and the occurrence of spontaneous seizures. Evidence obtained by Biagini et al. [78] now suggests that the duration of the latent period, as well as P450scc induction, is indeed increased in animals that have endured prolonged pilocarpine-induced SE. However, when neurosteroid synthesis is blocked with finasteride, the duration of the latent period is decreased [78], suggesting that by increasing GABA-mediated inhibition, neurosteroids can delay the development of a chronic epileptic condition, as was proposed before in the kindling model of epileptogenesis [79]. The same group then performed further analyses of P450scc immunoreactivity in the hippocampus of pilocarpine-treated rodents and found that it is expressed in several glial cells including astrocytes, oligodendrocytes and microglia [80]. In the same study, they also found that inhibition of neurosteroids with a daily administration of finasteride between day 4 and day 28 after SE could accelerate the development of spontaneous seizures, but only in animals that experienced an SE that lasted for more than 3 h and that exhibited significant increases of P450scc induction. In line with these findings, Joshi et al. [81] used the lithium-pilocarpine model to demonstrate that a single administration of finasteride on the fourth day after a 2 h SE is sufficient to decrease the duration of the latent period; according to these authors, this suggests that inhibition of neurosteroids must be performed at the time of down-regulation of δ-GABA receptor expression, which occurs between SE and the onset of spontaneous seizures.

**Figure 4:**
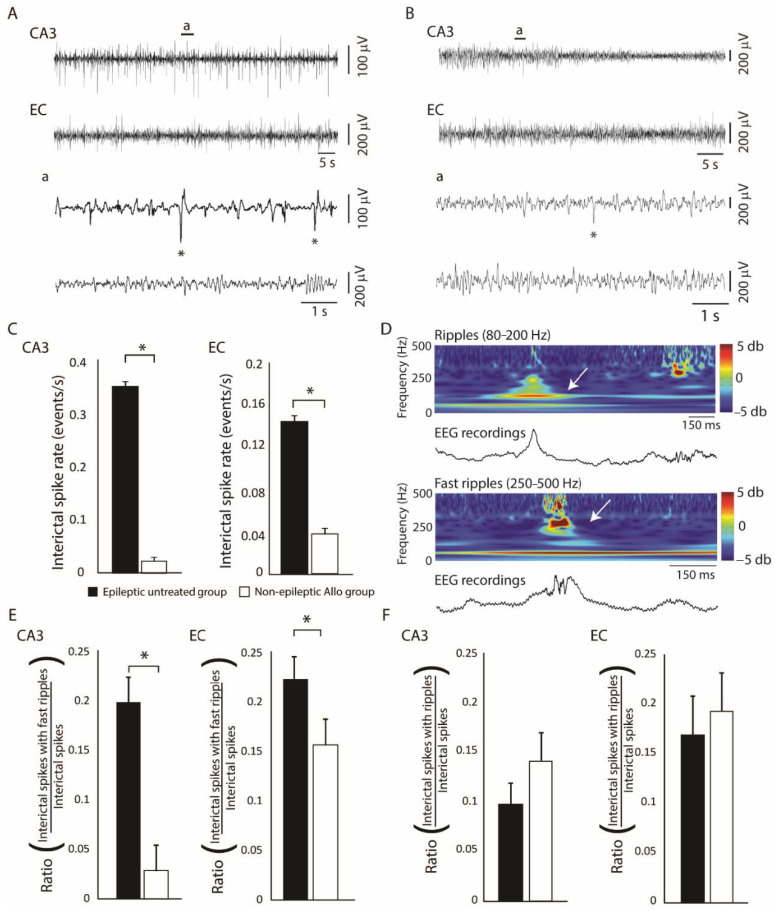
(**A**): Representative EEG recordings from the CA3 region of the hippocampus and the entorhinal cortex (EC) of an epileptic untreated animal. (**B**): Representative recordings in an epileptic animal that was treated with allopregnanolone. Insets (**a**) show interictal spikes (asterisks) on a lower timescale. (**C**): Bar graphs showing rates of interictal spikes in the epileptic untreated and non-epileptic allopregnanolone-treated group. Rates of interictal spikes in CA3 and EC were significantly lower in the non-epileptic allopregnanolone-treated group compared to the epileptic untreated group (* *p* < 0.05). (**D**): Example of a ripple (80–200 Hz) and a fast ripple (250–500 Hz) that occurred in association with an interictal spike, in an epileptic untreated animal. The wavelet analysis is shown to illustrate ripples and fast ripples (white arrows). (**E**): Bar graph showing ratios of interictal spikes with fast ripples on the total number of interictal spikes. In both CA3 and EC, rates of interictal spikes with fast ripples occurred at significantly lower rates in the non-epileptic allopregnanolone-treated group compared to the epileptic untreated group (* *p* < 0.05). (**F**): Bar graph showing ratios of interictal spikes with ripples on the total number of interictal spikes. No significant differences were observed between groups. Panels were modified from the study of Lévesque et al. [14].

Since allopregnanolone is a positive allosteric modulator of synaptic and extrasynaptic modulator of GABA_A_ receptors, its administration could functionally reverse the GABAergic deficit that may occur after SE. Lévesque et al. [14], therefore, assessed whether its administration during the latent phase could modulate spontaneous seizures in the pilocarpine rat model of MTLE. In their study, allopregnanolone was administered with a subcutaneous pump for 12 consecutive days starting 1 day after SE. Compared to the untreated group, in which 67% of them became epileptic, only 29% of allopregnanolone-treated animals were epileptic. These anti-epileptogenic effects were associated to significantly lower rates of interictal spikes in the CA3 and entorhinal cortex of non-epileptic allopregnanolone-treated animals compared to the untreated group (Figure 4A–C). Rates of interictal spikes with fast ripples (Figure 4D) were also significantly lower in the allopregnanolone-treated group compared to the untreated group (Figure 4E), whereas rates of interictal spikes with ripples (Figure 4D) were not significantly different between groups (Figure 4F). Interestingly, the effects induced by allopregnanolone were similar to those observed with the anti-epileptic drugs levetiracetam and lacosamide in the pilocarpine model of MTLE [82,83], confirming the potential of GABA_A_ receptor modulating neurosteroids in the treatment of epilepsy.

Endogenous levels of allopregnanolone and pregnanolone in the brain of rats that have been treated with kainic acid to induce an SE are also significantly modulated by spontaneous seizures. Lucchi et al. [84] recently observed reduced levels of allopregnanolone and pregnanolone in the hippocampus of epileptic animals 9 weeks after SE. Moreover, linear regression analyses revealed a significant positive correlation between endogenous levels of allopregnanolone and seizure frequency in the hippocampus (higher seizure rates of seizures were associated with increased levels of allopregnanolone), whereas this correlation was not observed in the neocortex. No significant correlations were observed between endogenous levels of pregnanolone and the frequency of spontaneous seizures and no alterations in circulating levels of allopregnanolone and pregnanolone were observed. It was, therefore, hypothesized that focal allopregnanolone synthesis in the hippocampus is increased in the presence of seizures as a compensatory mechanism, but that it fails in restoring levels of this neurosteroid.

## 4. Human Studies

The possible therapeutic use of neurosteroids for epilepsy was first explored in pediatric patients treated with the methylated analog of allopregnanolone, ganaxolone [85]. These authors examined 15 patients within the range of 5–15 years of age, suffering from drug-resistant epilepsy of various types; eight of them concluded the trial, at the end of which beneficial effects were observed in six patients. This pilot study was then followed by another one performed in adults with partial-onset seizures that were poorly controlled by antiepileptic drugs [86]. In this study, 131 patients completed the protocol (86 maintained on ganaxolone) and a reduction of about 11% in the seizure frequency was observed with ganaxolone. The most common adverse events were dizziness, fatigue and somnolence, found in approximately 15% of cases. Additionally, in view of the reduction in allopregnanololone and other steroid levels in patients with protocadherin 19 (PCDH19)—female limited epilepsy [87], trial is currently ongoing to evaluate if ganaxolone may be beneficial in patients with this rare neurological disorder [88]. However, caution on the effectiveness of the use of allopregnanolone analogues has been raised by a study on post mortem neocortical tissue obtained from children with epilepsy, in which authors found an altered composition of GABA_A_ receptors leading to reduced sensitivity to neurosteroids [89]. Therefore, these results suggest that children could be unresponsive to allopregnanolone analogues.

Unsatisfactorily, the allopregnanolone analog brenaxolone has been tested in patients with refractory or super-refractory SE [90]. After having successfully passed a phase I/II trial [91], a phase III trial did not find any beneficial effect of brexanolone (reported in Rossetti et al. [90]). However, Meletti et al. [92,93] found a marked reduction in progesterone, 5α-dihydroprogesterone, allopregnanolone and pregnanolone levels in the cerebrospinal fluid obtained by patients with SE, thus suggesting that administration of allopregnanolone or the analogs alone could be insufficient to reach an adequate compensation for the reported defect in various neurosteroid levels. In this regard, a more effective approach to re-establish normal levels of anticonvulsant neurosteroids should be made available. Such a goal could be possibly achieved by using trilostane, an inhibitor of peripheral conversion of pregnenolone to progesterone, which was found to increase the cerebral levels of different neurosteroids [94,95]. Recently, pretreatment with trilostane was shown to increase the neocortical and hippocampal levels of pregnenolone, progesterone, 5α-dihydroprogesterone and allopregnanolone, leading to the reduction in convulsive seizure duration in rats with kainic acid-induced SE [96].

Finally, progesterone is known to be effective in catamenial epilepsy, i.e., epileptic women who experience perimenstrual seizure exacerbation [97]. This effect could be related to the conversion of progesterone to allopregnanolone, as suggested by a case report in which a woman, cured with progesterone, developed refractoriness to antiepileptic drugs when she was treated with finasteride [98]. However, the appearance of resistance to antiepileptic drugs has been also reported in a woman who was treated with finasteride but did not receive progesterone as an add-on drug. This finding suggests that allopregnanolone levels might influence the response to the antiepileptic treatment [99].

## 5. Conclusions

We have reviewed here some experimental and clinical studies that were aimed at establishing the involvement of neurosteroids in the context of focal epileptic disorders. Clearly, the modulatory effects exerted by these compounds depends on their class (e.g., sulfated vs. nonsulfated), the subunit composition of the neurotransmitter receptors as well as the pathophysiological mechanisms that underlie specific epileptic disorders. For instance, much of the experimental evidence suggests that the ability of neurosteroids to influence neuronal excitability resides primarily in the enhancement of GABA_A_ receptor function. On the other hand, this physiological characteristic may result in opposite effects, depending on the contribution of GABAergic mechanisms to epileptiform synchronization [100]. Finally, it remains to be firmly established whether neurosteroids or their analogues can prevent the development of a chronic epileptic condition (i.e., whether they can successfully halt epileptogenesis).

## Abbreviations

4AP	4-aminopyridine
P450scc	P450 cholesterol side chain cleavage
HFOs	high-frequency oscillations
MTLE	mesial temporal lobe epilepsy
NEC	non-epileptic control
PCDH19	Protocadherin 19
PTX	picrotoxin
SE	status epilepticus
THDOC	Allotetrahydrodeoxycorticosterone
